# Impact of new antifungal medications on onychomycosis prescriptions and costs in Japan: A nationwide claims database study

**DOI:** 10.1111/1346-8138.17393

**Published:** 2024-08-08

**Authors:** Hideaki Miyachi, Daisuke Sato, Kentaro Sakamaki, Yaei Togawa, Kensuke Yoshimura

**Affiliations:** ^1^ Center for Next Generation of Community Health Chiba University Hospital Chiba‐shi Chiba Japan; ^2^ Department of Dermatology Chiba University Hospital Chiba‐shi Chiba Japan; ^3^ Department of Emergency Medicine Massachusetts General Hospital, Harvard Medical School Boston Massachusetts USA; ^4^ Hospital and Health Administration, Fujita Health University Graduate School of Medicine Toyoake Aichi Japan; ^5^ Faculty of Health Data Science, Juntendo University Urayasu Chiba Japan

**Keywords:** antifungal agents, claims data, economic burden, NDB Open Data Japan, onychomycosis

## Abstract

Onychomycosis, a fungal nail infection, is a common dermatological condition in Japan, with a prevalence of approximately 5%–10%. Despite the introduction of new antifungal medications and updated treatment guidelines published in 2019, data on real‐world prescription trends and the associated medical costs are limited. This study aimed to investigate the prescription patterns and medical costs of topical and oral antifungal medications for onychomycosis in Japan from fiscal years 2014 to 2021 using the National Database of Health Insurance Claims and Specific Health Checkups of Japan Open Data. We analyzed the annual prescription volumes and medical costs of four antifungal medications: efinaconazole, luliconazole, fosravuconazole, and terbinafine. The prescription volume of efinaconazole, a topical medication launched in 2014, rapidly increased and dominated the market share. Fosravuconazole, an oral medication introduced in 2018, showed an increasing trend, coinciding with a decline in efinaconazole prescriptions. Terbinafine, a well‐established oral medication, experienced a substantial decrease in prescription volume. The sex‐ and age‐adjusted prescription volume per 100 000 population was higher among older adults, particularly for efinaconazole. The total medical costs for onychomycosis treatment more than doubled in fiscal year 2015 compared with that for 2014, mainly driven by efinaconazole prescriptions, and exceeded 30 billion Japanese yen in fiscal years 2019–2021. The costs slightly decreased in fiscal years 2020 and 2021, possibly due to the introduction of fosravuconazole. The predominance of topical prescriptions, especially in older adults, raises concerns regarding adherence to the Japanese guidelines that recommend oral antifungals as the first‐line treatment for onychomycosis. The substantial increase in medical costs also highlights the economic burden of onychomycosis and the need for cost‐effective treatment strategies. This study provides valuable insights into the real‐world prescription trends and medical costs of onychomycosis treatment in Japan, suggesting an opportunity to assess potential gaps between guideline recommendations and clinical practice.

## 1 INTRODUCTION

Onychomycosis, a superficial fungal infection of the nail primarily caused by dermatophytes, affects millions of people worldwide.^1^, ^2^ In Japan, the estimated prevalence of onychomycosis is approximately 5%–10%,^3^, ^4^ with higher prevalence among older adults, who are more susceptible than younger individuals owing to diminished blood circulation, longer exposure to pathogenic fungi, and the presence of comorbidities such as diabetes mellitus and peripheral vascular disease.^5^ Despite the various treatments available, including topical and oral antifungals, the management of onychomycosis remains complex owing to the high recurrence rate and challenges associated with long‐term medication adherence.^6^ In clinical practice, topical medications were often used as the first‐line treatment regardless of the disease severity, according to a web‐based survey in 2016.^7^ However, low adherence rates and high recurrence rates have been reported with topical therapy.^8^

Over the past decade, several new treatments specifically indicated for onychomycosis have been introduced in Japan. Two topical antifungal medications, efinaconazole (Clenafin®; Kaken Pharmaceutical Co., Tokyo, Japan) and luliconazole (Luconac®; Sato Pharmaceutical Co., Tokyo, Japan), were launched in 2014 and 2016, respectively. Additionally, fosravuconazole (Nailin®; Sato Pharmaceutical Co.), an oral antifungal medication, was introduced in 2018. The 2019 Japanese Dermatological Association guidelines for the management of dermatomycosis^3^ recommend oral treatment (grade A recommendation) over topical treatment (grade B recommendation) based on the higher cure rate of oral treatment. This guidelines state that topical treatment can be considered for patients who are intolerant of oral medications due to hepatic dysfunction, mild‐to‐moderate onychomycosis severity, and patient preferences. Similar treatment approaches have been outlined in the guidelines of Western countries.^9^, ^10^

Two recent studies have reported web‐based surveys of prescription preferences of dermatologists for onychomycosis.^11^, ^12^ Both studies showed an increase in the appropriate use of oral medications, yet also indicated that a considerable number of topical medications continue to be prescribed for patients with onychomycosis who are suitable for oral medications. Despite these valuable insights, no study has examined nationwide prescription trends in Japan, where primary care physicians manage a significant number of patients with dermatological disorders.

This study aimed to address the gap between the recommended antifungal treatments for onychomycosis and actual prescriptions in Japan. Using data from the National Database of Health Insurance Claims and Specific Health Checkups of Japan (NDB), this study analyzed prescription trends of antifungal treatments for onychomycosis from fiscal years 2014 to 2021. By examining the prescription patterns of both topical and oral antifungal medications, we investigated the impact of newly introduced medications on the trends, assessed the alignment of treatments with current guidelines, and evaluated the associated medical costs.

## 2 METHODS

### 2.1 Data source

We used the NDB Open Data Japan for this study. The NDB is a comprehensive database containing data on electronic health insurance claims and specific health checkups across Japan, maintained by the Ministry of Health, Labour and Welfare of Japan (MHLW). It covers over 98%–99% of the Japanese population and includes information on patient demographics, diagnoses, procedures, and prescriptions.^13^, ^14^ The NDB has been widely used in various epidemiological studies and health services research in Japan,^15^ and is distributed for secondary use in four formats. Among the four formats, in this study we used the NDB Open Data Japan, the publicly available version of NDB provided by the MHLW as preprocessed spreadsheets on the MHLW website. The first set of NDB Open Data Japan includes data for fiscal year 2014. The eighth set of NDB Open Data Japan, which is the newest data available as of May 2024, including data for fiscal year 2021, was published in July 2023. We accessed the NDB Open Data Japan website^16^ and downloaded spreadsheets entitled “Number of calculations by sex, and age group” under “prescription” for fiscal years 2014 to 2021.

### 2.2 Study medications and study period

We focused on four antifungal medications used to treat onychomycosis: two topical medications (efinaconazole and luliconazole) and two oral medications (fosravuconazole and terbinafine). Efinaconazole and luliconazole were chosen because they are newly launched topical antifungals specifically indicated for onychomycosis. We only included the 5% luliconazole formulation specifically indicated for onychomycosis (i.e., Luconac®), while other 1% luliconazole formulations such as ointments and lotions were excluded. Fosravuconazole (a new oral antifungal agent) and terbinafine (a well‐established oral antifungal agent) were also included. Itraconazole was excluded from the analysis as it is also indicated for other fungal infections and the prescription data in the NDB Open Data Japan are not linked with diagnoses. Although itraconazole pulse therapy is a specific prescription regimen for onychomycosis, the NDB Open Data Japan only provides summarized data, preventing us from extracting detailed information on intermittent prescriptions to determine whether itraconazole was prescribed as pulse therapy. The study period was from fiscal year 2014 to 2021 (i.e., April 1, 2014, to March 31, 2021).

### 2.3 Data analysis

We analyzed the annual prescription volumes of each study medication using NDB Open Data Japan. For topical and oral medications, prescription volumes were expressed as the number of grams prescribed and the number of tablets prescribed, respectively. Additionally, prescription volumes for fiscal years 2019 and 2021 were described by sex and 5‐year age groups. These two fiscal years were selected to assess the impact of the new treatments and guidelines. Moreover, for these fiscal years, the index of topical to oral prescription volumes was calculated by dividing the total costs of topical medications (efinaconazole and luliconazole) by the total costs of oral medications (fosravuconazole and terbinafine) for each age group. Furthermore, age‐ and sex‐adjusted prescription volumes for fiscal years 2019 and 2021 were calculated using population data obtained from the official website of the Statistics Bureau of Japan (https://www.stat.go.jp). We also calculated the total annual medical costs associated with each medication based on the prices listed in the NDB Open Data Japan. Analyses were conducted using R version 4.4.0 (R Foundation for Statistical Computing, Vienna, Austria) and figures were produced using ggplot2 version 3.5.1.

## 3 RESULTS

### 3.1 Prescription volumes

**FIGURE 1:**
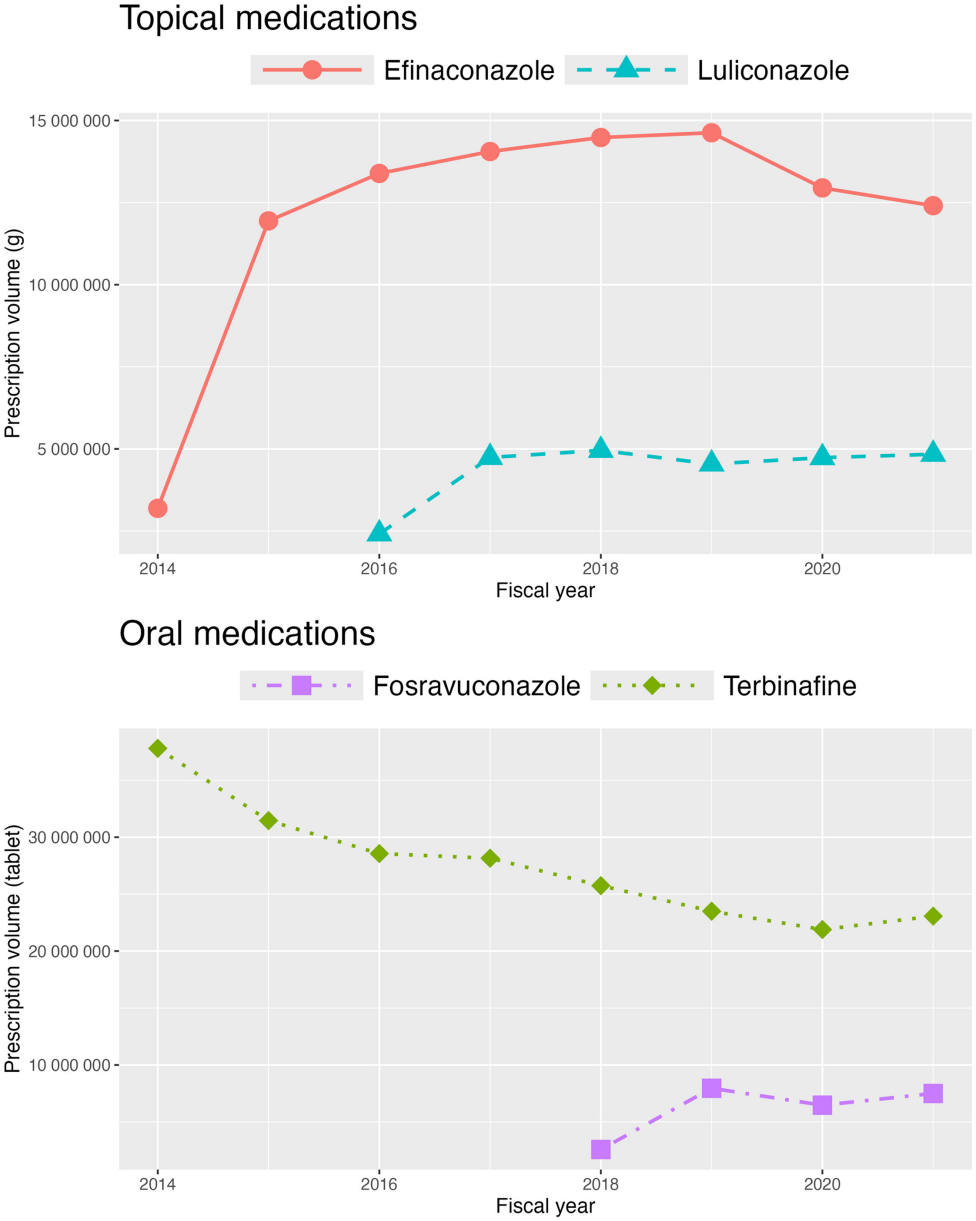
Annual prescription volumes of the study medications from fiscal years 2014 to 2021. Prescription volumes of topical medications are presented in grams, whereas those of oral medications are presented as the number of tablets prescribed.

The annual prescription volumes of the four study medications from fiscal years 2014 to 2021 are illustrated in Figure 1 and Supporting Information Table S1. The prescription volume of efinaconazole, launched in 2014, rapidly increased, reaching 11 942 033 g in fiscal year 2015, and continued to increase until it reached the highest level of 14 626 755 g in fiscal year 2019, before experiencing a slight decline in fiscal years 2020 and 2021. In contrast, the prescription volume of luliconazole, launched in 2016, was consistently lower than that of efinaconazole throughout the study period. Among the oral medications, the prescription volume of fosravuconazole, launched in 2018, displayed an upward trend, coinciding with a reduction in efinaconazole prescriptions. The prescription volume of terbinafine, a well‐established oral antifungal medication, experienced a substantial decrease from 37 799 008 tablets in fiscal year 2014 to 23 499 463 tablets in fiscal year 2019 and 23 067 940 tablets in fiscal year 2021.

**FIGURE 2:**
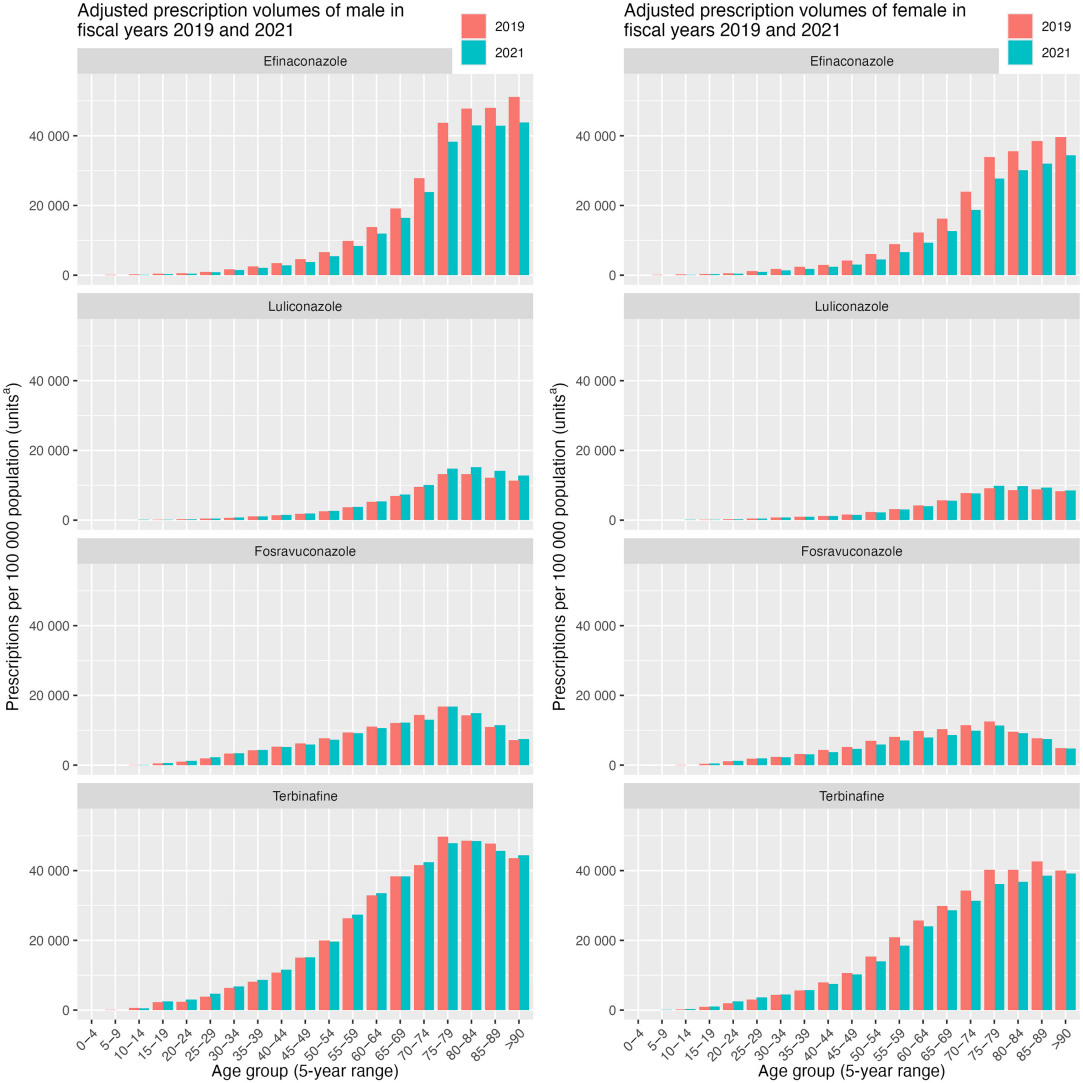
Prescription volumes per 100 000 population adjusted for sex and 5‐year age groups of males (left) and females (right) in fiscal years 2019 and 2021. Each medication is illustrated in a separate panel. ^a^Prescription volumes of topical medications (efinaconazole and luliconazole) are presented in grams, whereas those of oral medications (fosravuconazole and terbinafine) are presented as the number of tablets prescribed.

The distributions of prescription volumes in fiscal years 2019 and 2021 for each medication across 5‐year age groups are illustrated in Supporting Information Figure S1. The absolute number of prescriptions was the highest in the 70–79‐year age groups for all medications in both fiscal years. Topical medications were predominantly prescribed to elderly patients aged ≥65 years, whereas oral medications had a considerable number of prescriptions among younger age groups compared with topical medications (Supporting Information Figure S2). Additionally, the difference in prescription volumes of efinaconazole between fiscal years 2019 and 2021 was mainly attributed to the change in prescriptions among older patients. Regarding the difference between men and women, the absolute number of prescriptions for men was higher than that for women in most age groups, except for the 80+ years age groups. However, after adjustment for sex and age, the prescription volumes per 100 000 population were higher for men than for women in the 80+ years age groups as well (Figure 2). Notably, for efinaconazole, the sex‐ and age‐adjusted prescription volumes in the 80+ years age groups were comparable with those in the 70–79 years age groups, where the age‐ and sex‐adjusted prescription volumes were the highest. In contrast, prescriptions of oral medications were lower in patients aged ≥80 years, as indicated by the sex‐ and age‐adjusted analysis, for both fiscal years.

### 3.2 Medical costs

**FIGURE 3:**
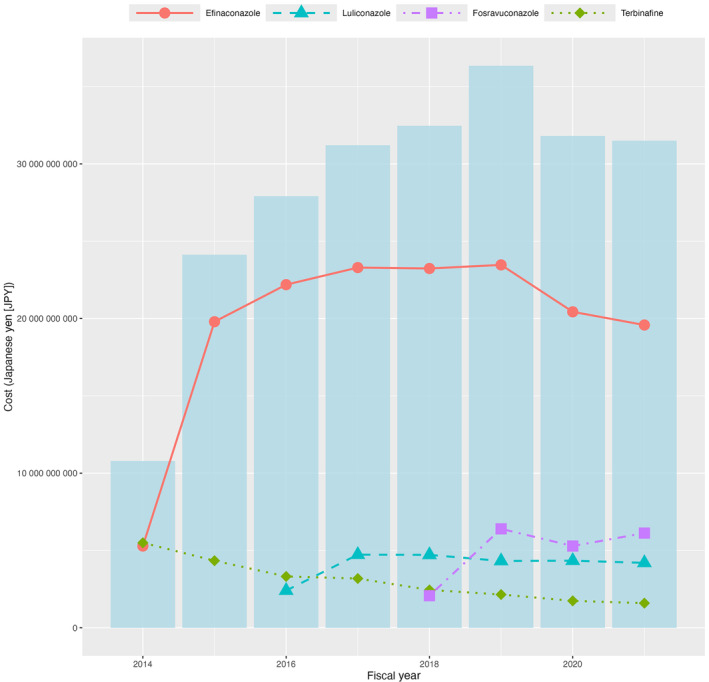
Annual medical costs associated with each medication and the total costs during fiscal years 2014 to 2021. The blue bars represent the annual total costs of the four medications.

The annual medical costs associated with each study medication, along with the total costs of all studied medications from fiscal years 2014 to 2021, are shown in Figure 3 and Supporting Information Table S2. Efinaconazole accounted for the highest proportion of medical costs among the four studied medications. The total cost of onychomycosis treatment medications sharply increased in fiscal year 2015, doubling from an initial amount of 10.8 billion Japanese yen (JPY) in fiscal year 2014 to 24.1 billion JPY in fiscal year 2015. This trend in the increase of medical costs continued, with the total cost peaking at 36.3 billion JPY in fiscal year 2019. Subsequently, there was a slight reduction in the total cost, decreasing to 31.8 billion and 31.4 billion JPY in fiscal years 2020 and 2021, respectively.

## 4 DISCUSSION

This study investigated the prescription trends of topical and oral antifungal medications for onychomycosis in Japan from fiscal years 2014 to 2021, using the NDB Open Data Japan. We observed a rapid increase in the prescription volume of efinaconazole, a topical antifungal medication, following its launch in 2014. Efinaconazole then dominated the market share among the studied medications. The prescription volume of fosravuconazole, an oral antifungal medication introduced in 2018, also showed an increasing trend, coinciding with a decline in efinaconazole prescriptions during fiscal years 2020 and 2021. Additionally, the prescription volume of terbinafine, a well‐established oral antifungal medication, showed a substantial decrease throughout the study period. These findings suggest that the introduction of new antifungal medications, particularly efinaconazole and fosravuconazole, significantly influenced the prescription patterns for onychomycosis treatment in Japan. The rapid increase in efinaconazole prescriptions may be attributed to its efficacy and favorable safety profile, as demonstrated in clinical trials.^17^, ^18^ The decline in terbinafine prescriptions could also have been attributable to the introduction of new treatments.

The predominance of topical antifungal prescriptions, especially efinaconazole, raises concerns about adherence to the Japanese Dermatological Association's guidelines,^3^ which recommend oral antifungal medications (grade A recommendation) as the first‐line treatment for onychomycosis. The guidelines state that topical antifungals should be considered for mild‐to‐moderate cases, patients who cannot tolerate oral medications, and those who prefer topical treatment. Similar recommendations have been outlined in the US^9^ and German^10^ guidelines. However, despite the publication of the Japanese Dermatological Association's guidelines, the prescription patterns in fiscal year 2019 (i.e., the year when the Japanese Dermatological Association's guidelines were published) and fiscal year 2021 (i.e., the most recent data available in the NDB Open Data Japan) remained similar, suggesting a potential gap between guideline recommendations and nationwide clinical practice.

The sex‐ and age‐adjusted prescription volumes per 100 000 population were higher in older adults than in the younger population, particularly for efinaconazole. This finding is consistent with the increased prevalence of onychomycosis in the older population due to factors such as diminished blood circulation, longer exposure to pathogenic fungi, and the presence of comorbidities.^6^ The high prescription volume of efinaconazole in older adults may reflect physicians' preference for topical medications in this population to avoid potential drug interactions and adverse effects associated with oral antifungals.^19^ However, it has been reported that the adherence and continuation rates for topical antifungals are not ideal, and they are often prescribed and continued without careful consideration.^12^ Thus, further optimization of the use of oral medications should be considered. Furthermore, recent studies on fosravuconazole in older adult patients have shown that oral treatments can be effective, with a frequency of adverse events similar to that in other age groups,^20^, ^21^ suggesting that more patients may benefit from treatment with oral medications.

The total medical costs for onychomycosis treatment with the studied medications more than doubled in fiscal year 2015 compared with that in fiscal year 2014, mainly driven by the increase in efinaconazole prescriptions. The total medical costs in fiscal years 2019 to 2021 exceeded 30 billion JPY, with efinaconazole accounting for the largest proportion, which highlights the economic burden of onychomycosis and the impact of new antifungal medications on healthcare costs. Our analysis showed that medical costs decreased in fiscal years 2020 and 2021 compared with that in fiscal year 2019. This decrease may be attributed to the publication of the guidelines in 2019^3^ and the introduction of fosravuconazole in 2018, which has higher efficacy and continuation rates than other treatments,^12^ resulting in an increased tendency of physicians to prescribe oral antifungals in accordance with the guidelines.^7^, ^12^ However, this study could not incorporate the additional medical costs associated with oral antifungals, such as blood tests. In Japan, a system for evaluating the cost‐effectiveness exists,^22^, ^23^ but medications for onychomycosis analyzed in this study do not fall under the criteria for official evaluation by the MHLW, therefore further research is needed to evaluate the cost‐effectiveness of different treatment strategies and to optimize resource allocation.

Recent studies have supported the cost‐effectiveness of oral medication for onychomycosis. A US study in 2016^24^ and its accompanying editorial^25^ highlighted that oral terbinafine offers higher efficacy with a considerably lower cost than efinaconazole, strongly supporting the cost‐effectiveness of oral medication over topical medication. More recently, another US study in 2020 modeling usage and estimation of the cost of medication for onychomycosis showed that efinaconazle is more expensive than oral treatment, even without considering relative efficacy.^26^ Furthermore, newer oral medications, e.g., fosravuconazole, have shown promising results in terms of efficacy and cost‐effectiveness.^27^ A study in 2023 from Japan^28^ reported that fosravuconazole was effective in patients with onychomycosis refractory to long‐term topical medication. Additionally, the average cost of oral fosravuconazole therapy was lower than that of topical medications, further supporting the cost‐effectiveness of oral medication.

The preference of topical medications among some practitioners may lie in the misconception (by both patients and physicians) that oral medications are dangerous.^25^ Recently, studies^20^, ^21^ have demonstrated the long‐term efficacy and safety of fosravuconazole in older patients, a population with a higher prevalence of onychomycosis^5^ and often considered at higher risk for adverse effects. These data consistently support the idea that oral medications, particularly with agents such as terbinafine and fosravuconazole, are significantly more cost‐effective than topical medications for the treatment of onychomycosis with an acceptable safety profile. While topical medications may have a role in certain cases, the evidence supports oral medications as the first‐line treatment for onychomycosis from both efficacy and cost‐effectiveness perspectives.

This study had some limitations. First, the NDB Open Data Japan does not link prescription data with diagnoses, which may have led to the inclusion of prescriptions for indications other than onychomycosis, therefore we focused on antifungal medications specifically indicated for onychomycosis treatment, such as efinaconazole, luliconazole (i.e., Luconac®), and fosravuconazole. Second, the lack of information on disease severity and treatment outcomes in the NDB Open Data Japan limits the interpretation of the appropriateness of the prescribing patterns of topical and oral medications. Thus, data from this study should be interpreted in conjunction with other studies that incorporate clinical data from electronic health records or surveys. For example, a recent web‐based questionnaire survey showed that subtypes, such as total and dystrophic onychomycosis, proximal subungual onychomycosis, as well as moderate‐to‐severe distal and lateral subungual onychomycosis, comprised 65.2% of the patients treated for onychomycosis.^11^ These data together may underscore the gap between guideline recommendations and clinical practice. Third, the inability to calculate the defined daily dose of topical medications hindered the direct comparison of prescription volumes between topical and oral antifungals. To address this limitation, we focused on the trends for each medication. Fourth, the coronavirus disease 2019 pandemic in 2020 and 2021 may have influenced patients' healthcare‐seeking behaviors and physicians' prescription patterns.^29^ Fifth, 2 years may not have been long enough to assess the impact of the new guideline publications and new oral treatments. Consequently, although there was a trend indicating an increase in the use of oral medications in accordance with the guidelines, this study may have underestimated this shift. Further research over a longer period is necessary to accurately capture these trends and the long‐term impact of guideline adherence on treatment practices. Despite these limitations, this study provides valuable insights into recent trends in onychomycosis treatment in Japan after the introduction of new treatments and guidelines published in 2019. The use of the comprehensive NDB Open Data Japan, which covers over 98%–99% of the Japanese population, strengthens the generalizability of our findings. Furthermore, although this study did not distinguish between prescriptions by dermatologists or non‐dermatologists, our study, which included prescriptions regardless of physicians' specialties, provides a comprehensive overview of the nationwide prescription trends.

In conclusion, this study highlights the dynamic shifts in prescription patterns for onychomycosis treatment, particularly the rapid introduction of new antifungal medications, such as efinaconazole and fosravuconazole, which have significantly altered treatment approaches. Despite the guideline recommendations for oral medications, the predominance of topical treatments in older adults suggests a potential gap between guideline recommendations and clinical practice. Furthermore, the substantial economic impact of onychomycosis treatments, particularly newer antifungal medications, necessitates the assessment of their cost‐effectiveness.

## CONFLICT OF INTEREST STATEMENT

None declared.

## ETHICS STATEMENT

As this study used publicly available NDB Open Data Japan, it was exempt from institutional review board approval.

## Supporting information


Appendix S1.
