# Supplementary material for: Impact of new antifungal medications on onychomycosis prescriptions and costs in Japan: A nationwide claims database study
Source: J Dermatol. 2024 Aug 8;51(9):1172–9. doi: 10.1111/1346-8138.17393 (PMC11483898; doi:10.1111/1346-8138.17393)
Supplement: Supplementary file 1 — Appendix S1. [file JDE-51--s001.docx]

**Impact of New Antifungal Medications on Onychomycosis Prescriptions and Costs in Japan: A Nationwide Claims Database Study**

Hideaki Miyachi, Daisuke Sato, Kentaro Sakamaki, Yaei Togawa, Kensuke Yoshimura

**Table S1**. Annual prescription volumes of the study medications from fiscal years 2014 to 2021

**Table S2.** Annual medical costs associated with each medication and the total costs for fiscal year 2014 to 2021 in Japanese Yen

**Figure S1.** Prescription volumes of males (left) and females (right) in fiscal years 2019 and 2021 of each medication by 5-year age groups

**Figure S2.** Index of topical to oral prescription volumes in fiscal years 2019 and 2021 by 5-year age groups

**Table S1.** Annual prescription volumes of the study medications from fiscal years 2014 to 2021

|  | Topical^a^ | |  | Oral^b^ | |
| --- | --- | --- | --- | --- | --- |
| Fiscal year | Efinaconazole  (Clenafin®) | Luliconazole  (Luconac®) |  | Terbinafine | Fosravuconazole  (Nailin®) |
| 2014 | 3,189,734 | NA |  | 37,799,008 | NA |
| 2015 | 11,942,033 | NA |  | 31,456,038 | NA |
| 2016 | 13,387,636 | 2,411,830 |  | 28,561,167 | NA |
| 2017 | 14,054,565 | 4,739,596 |  | 28,153,584 | NA |
| 2018 | 14,481,994 | 4,954,951 |  | 25,744,289 | 2,579,289 |
| 2019 | 14,626,755 | 4,540,056 |  | 23,499,463 | 7,953,423 |
| 2020 | 12,947,147 | 4,733,412 |  | 21,891,778 | 6,470,594 |
| 2021 | 12,406,076 | 4,836,102 |  | 23,067,940 | 7,496,137 |

Brand name for efinaconazole, luliconazole, and fosravuconazole are shown in parentheses. NA represents the fiscal year before the medication was introduced in Japan.

^a^ Prescription volumes of topical medications are presented in grams prescribed.

^b^ Prescription volumes of oral medications are presented in the number of tablets prescribed.

**Table S2.** Annual medical costs associated with each medication and the total costs for fiscal year 2014 to 2021 in Japanese Yen.

|  | Topical | |  | Oral | |
| --- | --- | --- | --- | --- | --- |
| Fiscal year | Efinaconazole  (Clenafin®) | Luliconazole  (Luconac®) |  | Terbinafine | Fosravuconazole  (Nailin®) |
| 2014 | 5,286,984,186 | NA |  | 5,497,190,857 | NA |
| 2015 | 19,793,919,862 | NA |  | 4,338,811,417 | NA |
| 2016 | 22,190,006,533 | 2,406,524,450 |  | 3,320,373,999 | NA |
| 2017 | 23,295,441,555 | 4,729,168,762 |  | 3,186,649,385 | NA |
| 2018 | 23,236,358,703 | 4,716,122,706 |  | 2,448,007,142 | 2,075,295,929 |
| 2019 | 23,468,629,191 | 4,321,225,361 |  | 2,151,171,695 | 6,399,324,258 |
| 2020 | 20,434,481,419 | 4,339,118,407 |  | 1,742,034,542 | 5,287,122,357 |
| 2021 | 19,580,510,323 | 4,200,154,537 |  | 1,593,536,815 | 6,125,093,216 |

Brand name for efinaconazole, luliconazole, and fosravuconazole are shown in parentheses. NA represents the fiscal year before the medication was introduced in Japan.


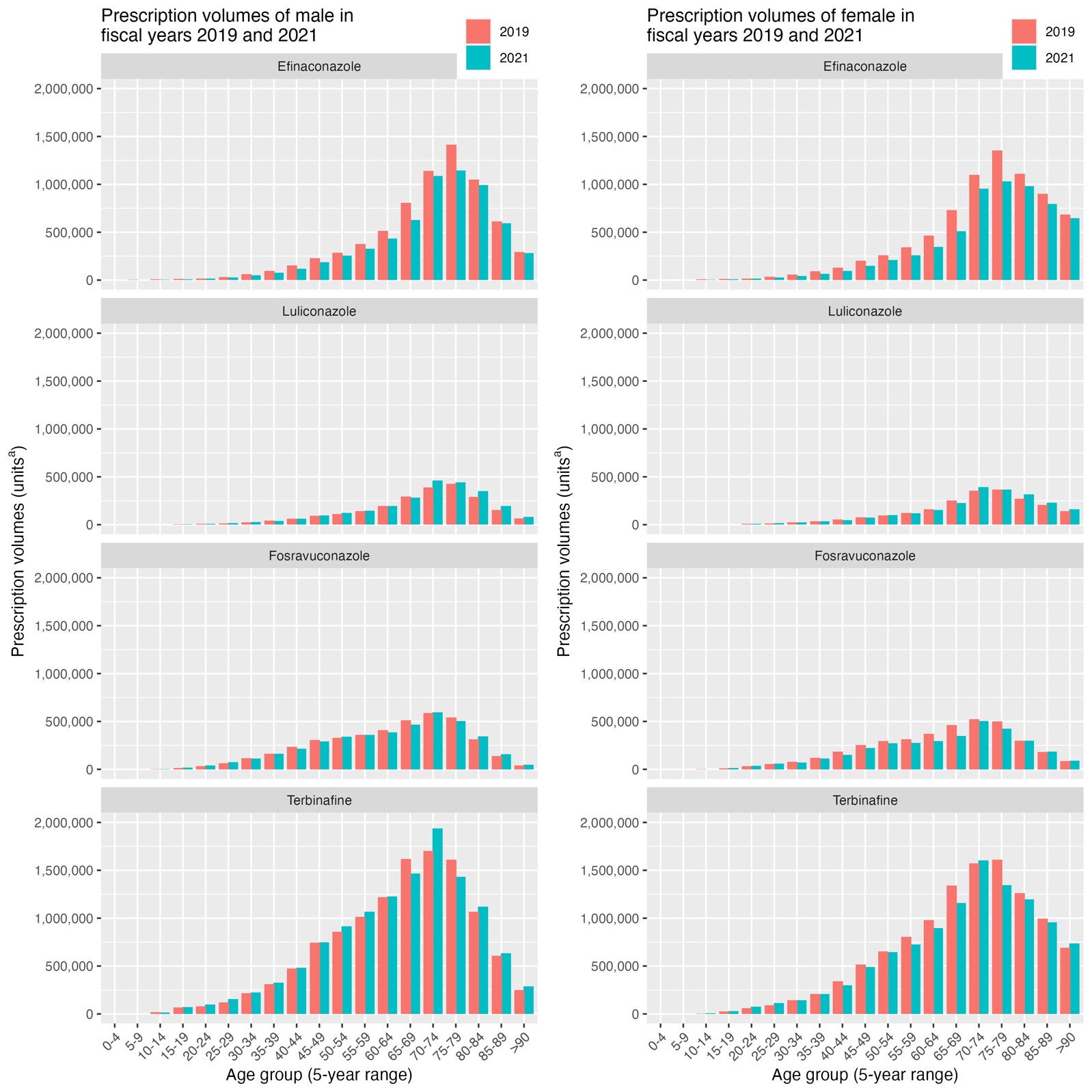


**Figure S1.** Prescription volumes of males (left) and females (right) in fiscal years 2019 and 2021 of each medication by 5-year age groups.

^a^Prescription volumes of topical medications (efinaconazole and luliconazole) are presented in grams, whereas those of oral medications (fosravuconazole and terbinafine) are presented as the number of tablets prescribed.

**
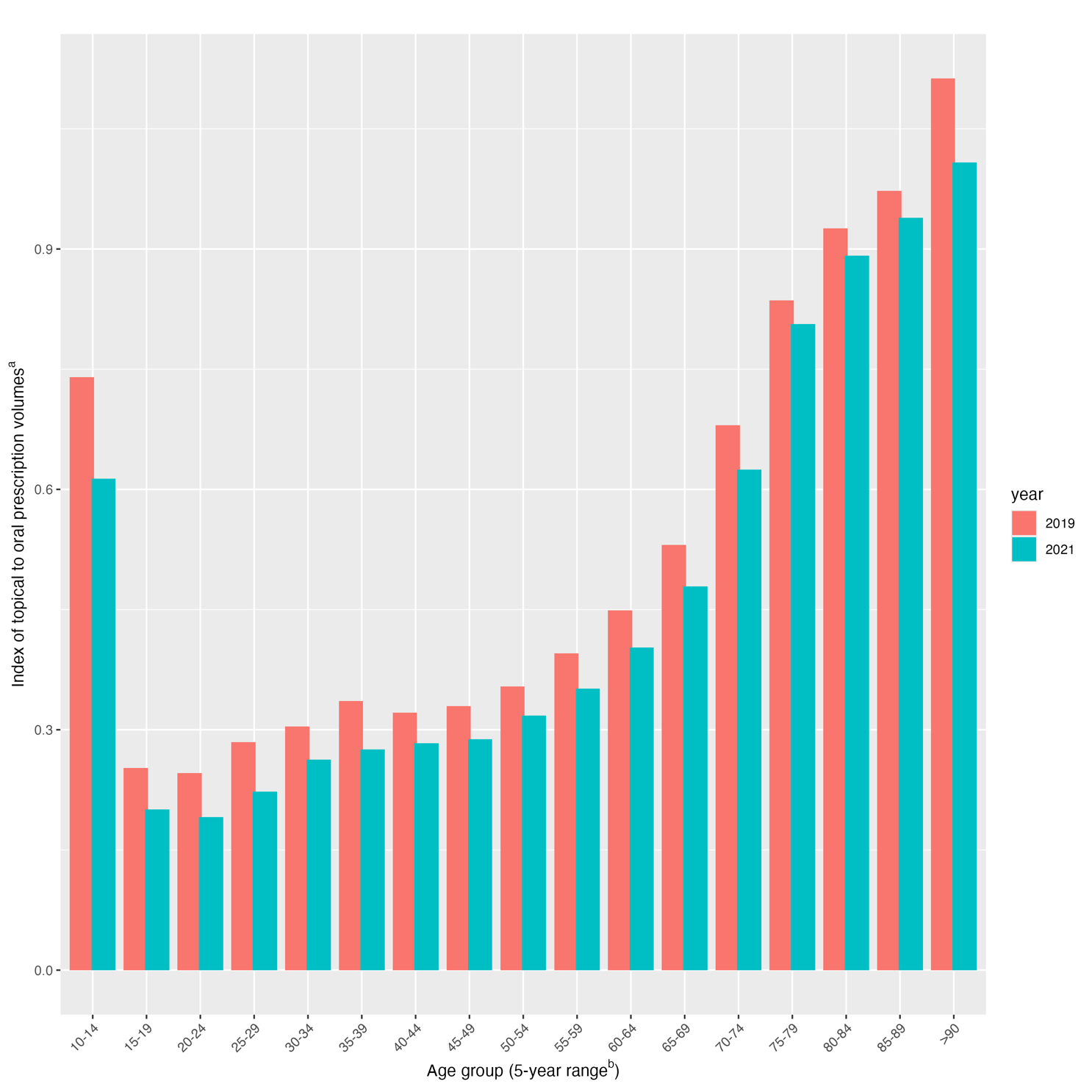
**

**Figure S2.** Index of topical to oral prescription volumes in fiscal years 2019 and 2021 by 5-year age groups. The index is calculated by dividing the total prescriptions of topical medications (efinaconazole and luliconazole) in gram by the total prescriptions of oral medications (fosravuconazole and terbinafine) in tablets for each age group. Higher bars indicate larger prescription volumes of topical medications than oral medications.

^a^The unit of measure for the index of topical to oral prescription volumes is gram per tablet.

^b^According to the publication criteria of NDB Open Data Japan, prescription volumes under specific thresholds are masked to protect privacy. Consequently, age groups 0–4 and 5–9 years were omitted from the plot owing to masked values.
